# Livestock producers' knowledge, attitude, and behavior (KAB) regarding antimicrobial use in Ethiopia

**DOI:** 10.3389/fvets.2023.1167847

**Published:** 2023-05-19

**Authors:** Takele B. Tufa, Fikru Regassa, Kebede Amenu, J. A. Stegeman, Henk Hogeveen

**Affiliations:** ^1^Department of Farm Animal Health, Faculty of Veterinary Medicine, Utrecht University, Utrecht, Netherlands; ^2^College of Veterinary Medicine and Agriculture, Addis Ababa University, Bishoftu, Ethiopia; ^3^Animal and Human Health Programme, International Livestock Research Institute (ILRI), Addis Ababa, Ethiopia; ^4^Business Economics Group, Wageningen University and Research, Wageningen, Netherlands

**Keywords:** antimicrobials use (AMU), antimicrobial resistance (AMR), knowledge–attitude–behavior (KAB) study, livestock producers, low-income country

## Abstract

**Introduction:**

Inappropriate antimicrobial use (AMU) in livestock production is an important aspect of the global burden of antimicrobial resistance (AMR). In Ethiopia, a low-income country with a large and increasing livestock population, AMU in food animals is not properly regulated. Hence, farmers are fully free to use antimicrobials to their (perceived) benefit. Therefore, understanding farmers' mindsets is important to improve antimicrobial stewardship in the livestock sector.

**Methods:**

This cross-sectional study was conducted to assess livestock disease management practices and knowledge, attitude, and behavior (KAB) among livestock producers regarding AMU, residues, and resistance, as well as factors potentially explaining differences in KAB. We determined the KAB of livestock owners of three selected districts of central and western Ethiopia (n = 457), using a pretested questionnaire administered through face-to-face interviews. Logistic regression was used to evaluate the association between potential explanatory variables and the KAB scores of the respondents.

**Results:**

The results showed that 44% of the farmers used antimicrobials in the past few years, where antibiotics (21%) and trypanocides (11%) were most widely used to manage livestock diseases. Furthermore, most farmers showed poor knowledge about AMU, residues, and AMR (94%) and unfavorable attitudes (<50% correct answers) toward contributing factors for AMR (97%). On the contrary, 80% of the respondents had overall good behavior scores (≥50% correct answers) related to AMU. Multivariate analysis results showed that having good knowledge, keeping ≥2 animal species, and the occurrence of ≥4 livestock diseases on the farm in a year were strong predictors of bad behavior scores (*p* < 0.05). The findings of the current investigation also revealed that the incidence of livestock diseases on the farm and a higher level of formal education significantly contributed to better knowledge and desirable attitudes but bad AMU behavior.

**Conclusion:**

A low level of awareness about and undesirable attitudes toward AMU and AMR could potentially affect farmers' behavior toward judicious AMU, thus requiring awareness creation efforts on livestock disease management practices.

## 1. Introduction

Globally, antimicrobials are widely prescribed in the livestock industry to treat and prevent infections (1, 2). Intensification and expansion of livestock production to meet the increasing demand for animal proteins are predicted to double the consumption of antimicrobials in the livestock sector in developing countries by 2030 (3, 4). However, antimicrobial use (AMU) in livestock production, as indicated in various studies, is considered an important driver of antimicrobial resistance (AMR) (5–7). As a consequence, indiscriminate use of antimicrobials in food animals has put the health of the public at risk (8–10) and can be detrimental to human and animal health and the environment.

Ethiopia has the largest livestock population in Africa (11), with a production level below the African average (12). The coexistence of different production systems and agroecological zones (AEZ) is suitable for harboring various pathogens that affect livestock health and production. To supply animal products to the growing population, the livestock sector has started to dramatically increase the production with a moderate to high level of intensification (13), which could increase antimicrobial consumption (3, 4). Moreover, the fast expansion of urban/peri-urban farms around populated cities will result in increased risks of pollution, inappropriate use of antibiotics, and outbreaks and transmission of zoonotic diseases (13, 14).

Studies on AMU in food animals are of paramount importance to understanding the potential risks posed by the emergence of AMR to animals and public health (9, 15). Further detailed understanding of AMU can pave the way to taking effective actions (16). In many high-income countries, monitoring and AMU stewardship programs are implemented (17). In low- and middle-income countries (LMICs), to date, there is relatively little known about AMU and the factors behind AMU. Several institutional challenges are experienced by LMICs in implementing AMU stewardship measures in animal production. Inconsistent policies governing AMU in animal production, the absence of AMU regulation that restricts access to critically important antimicrobials without prescription, and the lack of systematic post-market quality surveillance of veterinary antimicrobials are the most important challenges (18). In addition to those institutional challenges, prudent use of antimicrobials is also a matter of farmers' behavior, and it has been described that a lack of AMR awareness and risk perception is important (19).

Previous studies conducted in localized areas of Ethiopia revealed the presence of poor AMU practices among food animal-rearing communities (20–23). However, the information generated from the western parts of Ethiopia with an extensive farming system and diverse disease distribution is scarce, and the situation can vary from the central parts of the country. Hence, to establish proper AMU behaviors in the livestock sector of Ethiopia and other LMICs, this study assessed the farmers' livestock disease management practices and the contribution of knowledge and attitudes of livestock producers to their behaviors of AMU, antibiotic residues, and AMR. This is expected to help develop evidence-based policies to reduce AMR in livestock producers in LMICs in general and in Ethiopia in particular.

## 2. Materials and methods

### 2.1. Study area

**Figure 1:**
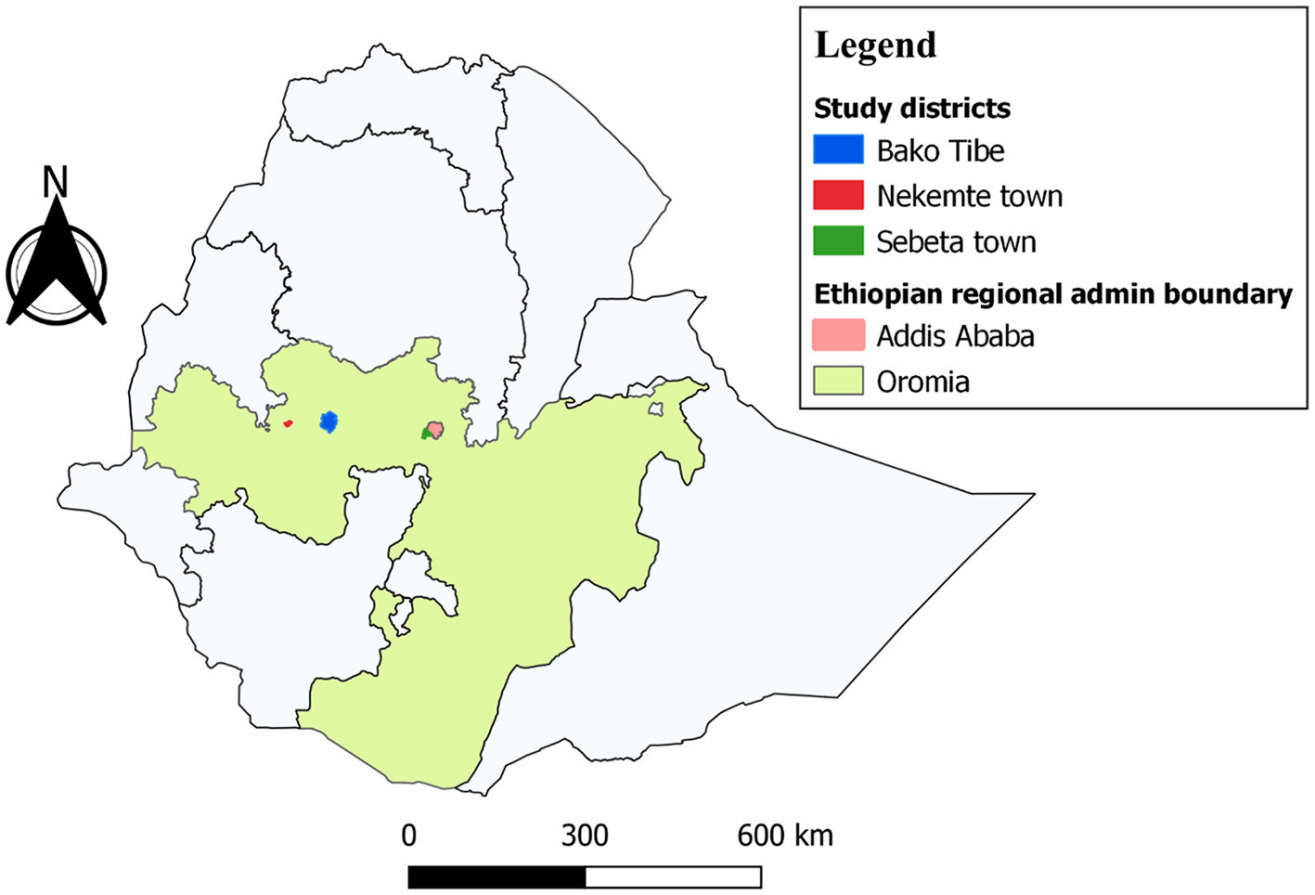
Map of the study area.

A questionnaire-based cross-sectional study was conducted between April and December 2018 involving livestock keepers in central and western Ethiopia. A total of three sites (see Figure 1) with various accessibility levels for veterinary services and variations in livestock disease distribution and production systems were purposely selected and included in the present study. The chosen sites were Sebata, Nekemte, and Bako districts and differ in their apparent accessibility for veterinary services. The variation in the distribution of livestock diseases, for instance, trypanosomiasis and respiratory diseases of contagious bovine pleuropneumonia (CBPP) or pasteurellosis, could potentially affect the AMU history on the farms.

Sebata (also called Sebeta or Sabbataa) is one of the urban districts in the Oromia Special Zone at 24 km from Addis Ababa (Figure 1). The town is in the central area of Ethiopia. The majority of farmers in this district are urban and peri-urban residents. The livestock keepers in this town are expected to have relatively good veterinary services and diagnostic facilities. Livestock diseases, blackleg, anthrax, foot and mouth disease (FMD), and pasteurellosis are mostly reported at the district veterinary clinic, while trypanosomiasis is absent. Bako is in the western border area of central Ethiopia in the west Showa zone of the Oromia region, ~251 km west of Addis Ababa. The majority of livestock producers are residing in peri-urban and rural areas of the district. Bako is characterized by various livestock diseases in which trypanosomiasis, pasteurellosis, sheep pox, anthrax, blackleg, coccidiosis, and rabies are quite common in the area (24). Nekemte is a city in the East Wollega zone of the Oromia regional state located 331 km from Addis Ababa in western Ethiopia (Figure 1). The subtropical climatic conditions of the city (25) favor various livestock diseases, including CBPP and trypanosomiasis. Most livestock owners reside in peri-urban and rural areas of the district and practice self-treatment of their animals with the frequent practice of AMU.

In all the surveyed districts, different livestock farming systems are present. Farmers living in the urban parts of the study areas practice intensive livestock farming in which the farmers buy feed and crop residues to feed their animals (26). The peri-urban and rural farmers practice a mixed crop-livestock farming system. The draft power and manure of the livestock are used to cultivate the crops, and the crop residues are used as feed for the animals.

### 2.2. Questionnaire design

The questionnaire design was based on the knowledge, attitude, and behavior (KAB) framework (27). The questionnaire included open-ended (*n* = 4) and closed (*n* = 47) questions about household demographics and farm characteristics (seven questions), livestock disease occurrence and management practices (nine questions), use of antibiotics (i.e., drugs which are used to treat bacterial infections) or antimicrobials (drugs that are used to treat or prevent infectious diseases caused by bacteria, fungi, protozoans, and viruses; it includes antibacterial, antifungal, antiviral, and antiprotozoal; 21 questions), antibiotic residues (six questions), and AMR (six questions; see details of the questionnaire in Supplementary material 1). Of these, the questionnaire included five knowledge, 13 attitude, and 15 behavior questions to assess the KAB of the respondents. The knowledge questions were “yes vs. no” and with requiring further explanations (where necessary). Similarly, the questionnaires about attitude and behavior were also prepared with “yes vs. no” questions.

The questionnaire was prepared in English using information obtained from our previous study conducted in central Ethiopia (20), and similar studies conducted elsewhere (27–30). Then, the questionnaire was translated into two commonly used local languages (Afan Oromo and Amharic) to use where necessary for collecting response information. Questionnaires were subjected to a piloting test of 20 household heads of dairy farms at Bishoftu town and revised for validation. As a result of this preliminary test, knowledge questions were redesigned for a minimal level of knowledge as the biological understanding of the participants regarding the details of antibiotics or antimicrobials turned out to be very low during pretesting of the questionnaire.

### 2.3. Data collection

Per selected study district, six kebeles (the smallest administrative units in Ethiopia) in and around the towns with small- to medium-scale livestock farmers were selected. Those kebeles were identified as urban, peri-urban, and rural communities (two from each were selected for each district). Depending on the number of livestock producers, 20–35 livestock producers were randomly chosen from each kebele.

The questionnaire was applied through a face-to-face interview by trained veterinarians and other animal health professionals working in the localities. Responses were, then, documented by translating them into English for further analysis. In the local context, the translated versions of the terms “antibiotics” and “antimicrobials” were used interchangeably. Most farmers were aware of the English word “antibiotics” but did not differentiate it from “antimicrobials.” Answers to open-ended questions were recorded and assessed for their correctness by asking respondents for further explanations. Each interview took on average 50 min to complete. In gathering information on which drugs are used on the farm, we followed a previously used protocol (21). Commonly available and used drugs at each study site were bought at the veterinary pharmacy or drug stores and put in a demonstration box to facilitate the interaction of enumerators with livestock owners. Furthermore, data collectors got information from community animal health workers, the district livestock bureau, and community leaders (farmers' delegates) about farmers' involvement in livestock production activities. Using the information provided, the person who was judged to have the most responsibility for livestock production in the household was invited by the enumerator for an interview.

A single proportion estimation was applied to determine the sample size required (31), with a 95% confidence interval, 5% margin of error, and an assumption that 50% (*p* = 0.5) of livestock producers have proper AMU in livestock production. The minimum sample size required was 384 producers; however, to collect sufficient data from three different sites, for potential incomplete responses and to increase the significance of the results, in the present field survey, 580 livestock owners (household level) were interviewed, of which 457 were completed.

Ethics approval for the study was granted through the Institutional Review Board of Addis Ababa University College of Veterinary Medicine and Agriculture (Ref. No: VM/ERC/01/06/10/2018). Participants were briefed about how their responses would be used and informed that they could withdraw from the study at any time. Verbal consent was sought and obtained from those who chose to proceed with the study.

### 2.4. Data management and analysis

Data acquired from the interviews were entered into a Microsoft Excel dataset, which was created specifically for the study using Microsoft Office 365. The data generated from the questions were reorganized to assess the KAB of respondents. The outcomes concerning knowledge questions were reclassified as “correct” when the response is “yes” and “incorrect” when the response is “no” or “I do not know.” For the attitude questions, when the response was “yes or agree” with a positive statement, it was classified as a “desirable” attitude. The reverse was considered an “undesirable” attitude. The responses to questions about farmers' behaviors were recorded as either “correct vs. wrong” or “good behavior vs. bad behavior.” Data were coded by giving 1 to “correct” or desirable answers and 0 to the “wrong/unanswered option” or undesirable response to a given question. Answers to open questions were also coded into categorical variables (knowledge: correct vs. incorrect; attitude: desirable vs. undesirable; behavior: good vs. bad). The data were inspected for data entry errors. Descriptive statistics were computed to describe household demographics and farm characteristics. The percentages of “appropriate” answers (i.e., correct answers in the knowledge question, favorable attitude in the attitude question, and application of proper management practices in the behavior section) were calculated for each KAB item.

The participants' overall score for KAB was categorized using a modified Bloom's cutoff point. A score between 80 and 100% was categorized as good, 50 and 79% as moderate, and <50% as poor, as applied by the study mentioned in Reference (32–34). The aggregate score for all knowledge questions would range from 0 to 5 points for a given participant. Participants' overall knowledge score was categorized as good (sufficient), moderate, and poor if the score was ≥4 points, 3 points, and <3 points, respectively. The attitude of farmers toward AMU, residues, and AMR could vary from 0 to 13 and was graded as favorable (good), less favorable (moderate), and unfavorable (poor) if the score was ≥11 points, 7–10 points, and <7 points, respectively. Similarly, the overall livestock producers' behavior score would range from 0 to 15 and was categorized as good, moderate, and bad if the score was ≥12 points, 8–11 points, and <8 points, respectively. The final KAB scores were dichotomized for further analysis and those answers ≥50% correct (score of both good and moderate) in a knowledge, attitude, and behavior section of the questionnaire were considered to have sufficient (good) knowledge, favorable attitudes, and good behavior while those answers <50% correct (score of poor) were considered to have insufficient (poor) knowledge, undesirable (unfavorable) attitudes, and bad behavior, respectively.

**Figure 2:**
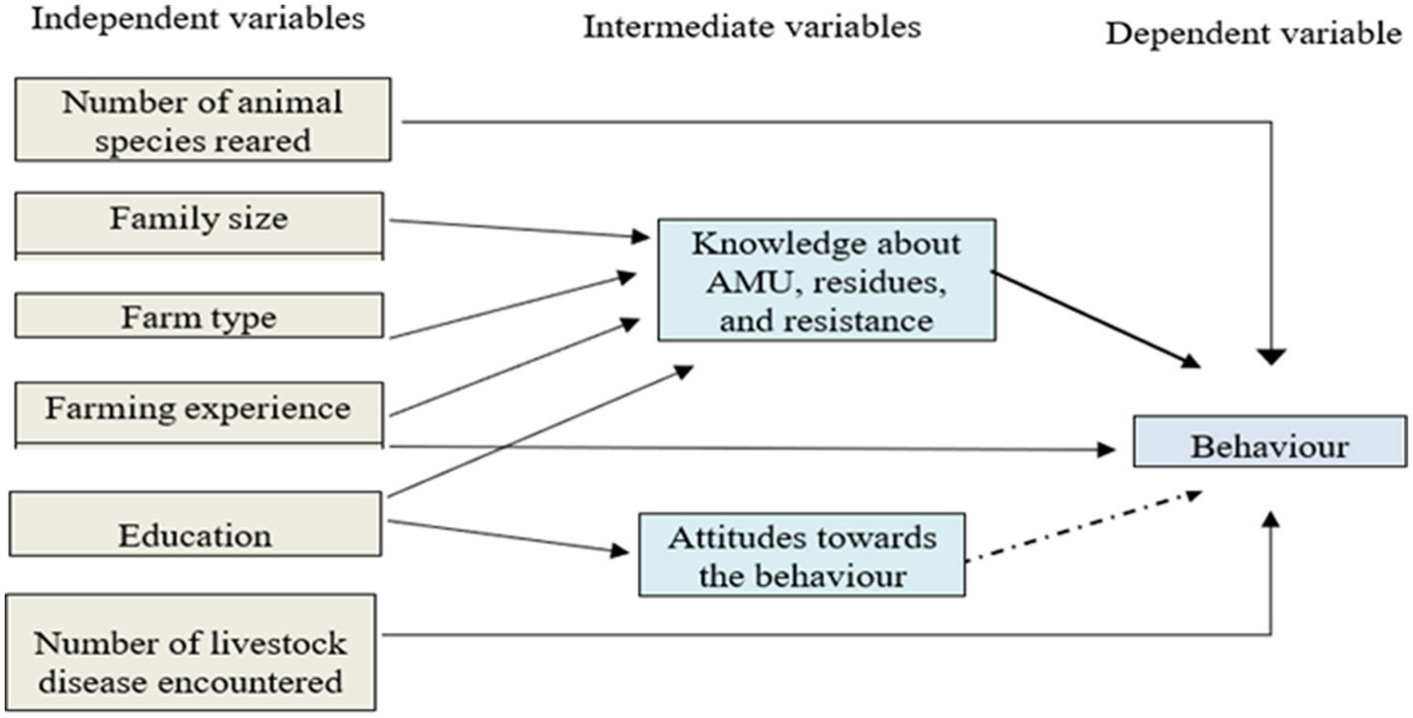
Schematic presentation of the model behavior, where the straight type black color arrow represents a dependence relationship in a multivariate analysis (*p*-value < 0.05) and a dash type black color arrow represents a correlational relationship in a univariate analysis.

The interaction of knowledge, attitude, and behavior is dynamic and can be affected by various factors (29). In this study, we constructed a conceptual framework (Figure 2) to estimate the nature and relationships that may influence the knowledge, attitude, and intention to carry out a particular behavior.

The influence of sociodemographic characteristics and livestock disease incidence at farm levels on participants' KAB scores was analyzed using logistic regression models. As a first step, a univariate analysis was carried out to determine factors associated with KAB scores. Before conducting the multivariable analyses, the variance inflation factor (VIF) was calculated to identify the presence of multicollinearity between the independent variables. No high levels of multicollinearity were observed between independent variables; all the VIFs were < 10, as applied by Sindato et al. (35).

To construct a final logistic regression model, first, the association between independent variables and each of the KAB domains was observed for statistical significance at a *p*-value of ≤ 0.25. From the univariate analysis, all statistically significant variables were included in the multivariate analysis. However, gender was not included in the model due to gender imbalance. Generalized linear models (GLMs) with a binomial-logit link function were fitted to the data to assess the effect of different predictors on the outcomes of interest (e.g., knowledge of farmers on AMU, residues, and AMR, attitude toward the good behavior, and behavior of farmers to apply appropriate AMU at their farms). A forward stepwise selection GLM was used to build the final multivariable regression models. Covariates with a *p*-value of < 0.05 were included in the final regression model. Attitude score was forced into the model to be considered as a predictor variable of the behavior. The final behavior model is presented in Figure 2.

The results of the univariate and multivariate logistic regression analyses were reported as odds ratio (OR) and adjusted odds ratio (OR_adjusted_), respectively, together with their 95% corresponding confidence intervals (CIs) at a *p*-value of 0.05. Spearman's rank-order correlation coefficient (*rho, r*) was run to describe the relationship and direction of the association among the KAB scores of the participants. Analysis was performed using STATA software version 17 (Texas, USA).

## 3. Results

### 3.1. Participants' demographics and farm characteristics

The sociodemographic characteristics of the surveyed livestock producers are presented in Table 1. The vast majority (85%) of the participants were male, were between 30 and 60 years old (78%), and had a minimum of 5 years of livestock farming experience (86%). Most of the respondents (61%) at least attended primary school; 42% completed only primary education or less, while 39% never attended formal school (Table 1). Of livestock reared by farmers in the study area, the type that most of the farmers keep was cattle (97%), followed by chicken (46%), sheep (13%), and goats (10%; Table 1). The findings on livestock herd size indicated that the majority of farmers were smallholders, where most of them keep ≤ 10 animals (cattle 81%, chicken 40%, goats 9%, and sheep 12.5%) in their farms (Table 1). Of the farmers who attended college diploma or university degree (6%), most of them (68%, 17/25) keep both cattle and chicken together.

**Table 1:** Sociodemographic characteristics of livestock producers surveyed concerning antimicrobial use, residues, and resistance.

**Variable**	**Parameters**		**Percent of participants (%)**			
			**Bako %**	**Nekemte %**	**Sebata %**	**Overall %**
			**(*****N*** = **185)**	**(*****N*** = **117)**	**(*****N*** = **155)**	**(*****N*** = **457)**
Gender	Female		20	7	16	15
	Male		80	93	84	85
Age group (years)	< 30		21	7	23	18
	30–45		57	80	49	60
	46–60		16	14	23	18
	>60		5	0	5	4
Education level	Illiterate (no formal education)		34	29	52	39
	Primary school		42	50	36	42
	High school		21	11	8	14
	College or university		4	10	4	6
Livestock farming experience (years)	< 5		14	2	12	10
	5–15		35	21	38	32
	>15		49	66	50	54
	Prefer not to answer		3	12	0	4
Household size (number of children)	0–3		11	19	19	16
	4–7		48	50	44	47
	7–10		37	22	34	32
	>10		2	1	3	2
	Prefer not to answer		2	8	0.0	3
Type of livestock kept	Cattle		97	100	97	98
	Chicken		41	73	32	46
	Sheep		17	12	9	13
	Goats		12	17	3	10
Herd size (number)	Cattle	1–5	49	15	45	39
		6–10	36	46	46	42
		11–20	10	35	5	14
		>20	2	2	1	1.8
	Chicken	1–5	21	39	16	24
		6–10	10	31	12	16
		11–20	8	3	3	4.6
		20–50	2	0	1	1.3
		>50	0	1	0.6	0.4
	Goats	1–10	11	17	2	9
		>10	1	0	1	0.7
	Sheep	1–10	16	12	8	12.5
		>10	1	0	1	0.7

### 3.2. Livestock disease management practices

The assessment conducted on farmers' awareness regarding livestock diseases showed that blackleg was mentioned most frequently (62%), followed by trypanosomiasis (42%) and anthrax (27%). Farmers reported the occurrence of a large number of infectious diseases in the year before the interview (2017/2018) on their farms: blackleg (63%), FMD (60%), pneumonic pasteurellosis (57%), anthrax (50%), and trypanosomiasis (49%; Table 2). The farmers' reported occurrence of disease differed statistically significantly (*p* < 0.05) between the study districts.

**Table 2:** Livestock diseases encountered on 457 farms in central and western areas of Ethiopia as reported by respondents (between September 2017 to August 2018).

**What are livestock diseases often encountered on your farm?**	**Bako**	**Nekemte**	**Sebata**	**Total**
	**(*N* = 185)**	**(*N* = 117)**	**(*N* = 155)**	**(*N* = 457)**
	**% (** ***n*** **) of respondents**	**% (** ***n*** **) of respondents**	**% (** ***n*** **) of respondents**	**% (** ***n*** **) of respondents**
Bloat	62 (115)	78 (91)	65 (100)	67 (306)^a^
Blackleg	64 (119)	97 (114)	37 (57)	64 (290)^a^
Diarrhea	63 (116)	53 (62)	72 (112)	64 (290)^a^
FMD	62 (115)	39 (46)	74 (115)	60 (276)^a^
Pasteurellosis	64 (118)	66 (77)	43 (67)	57 (262)^a^
Mastitis	62 (114)	89 (104)	28 (43)	57 (261)^a^
Anthrax	63 (116)	46 (54)	37 (58)	50 (228)^a^
Trypanosomiasis	68 (126)	86 (100)	0 (0)	50 (226)^a^
LSD	62 (115)	40 (47)	23 (35)	43 (197)^a^
CBPP	62 (114)	34 (40)	78 (121)	41 (188)^a^
Bovine TB	62 (114)	28 (33)	14 (21)	37 (168)^a^
Others^*^	1 (1)	14 (16)	0 (0)	4 (17)^a^

N, number of respondents surveyed; %, percent of the count who answered “yes;” n, frequency of count who answered “yes.”

* “Others” indicates ectoparasites, lungworm, and ringworm.

FMD, foot and mouth disease; CBPP, contagious bovine pleuropneumonia.

a Indicates p-value of ≤ 0.001.

**Figure 3:**
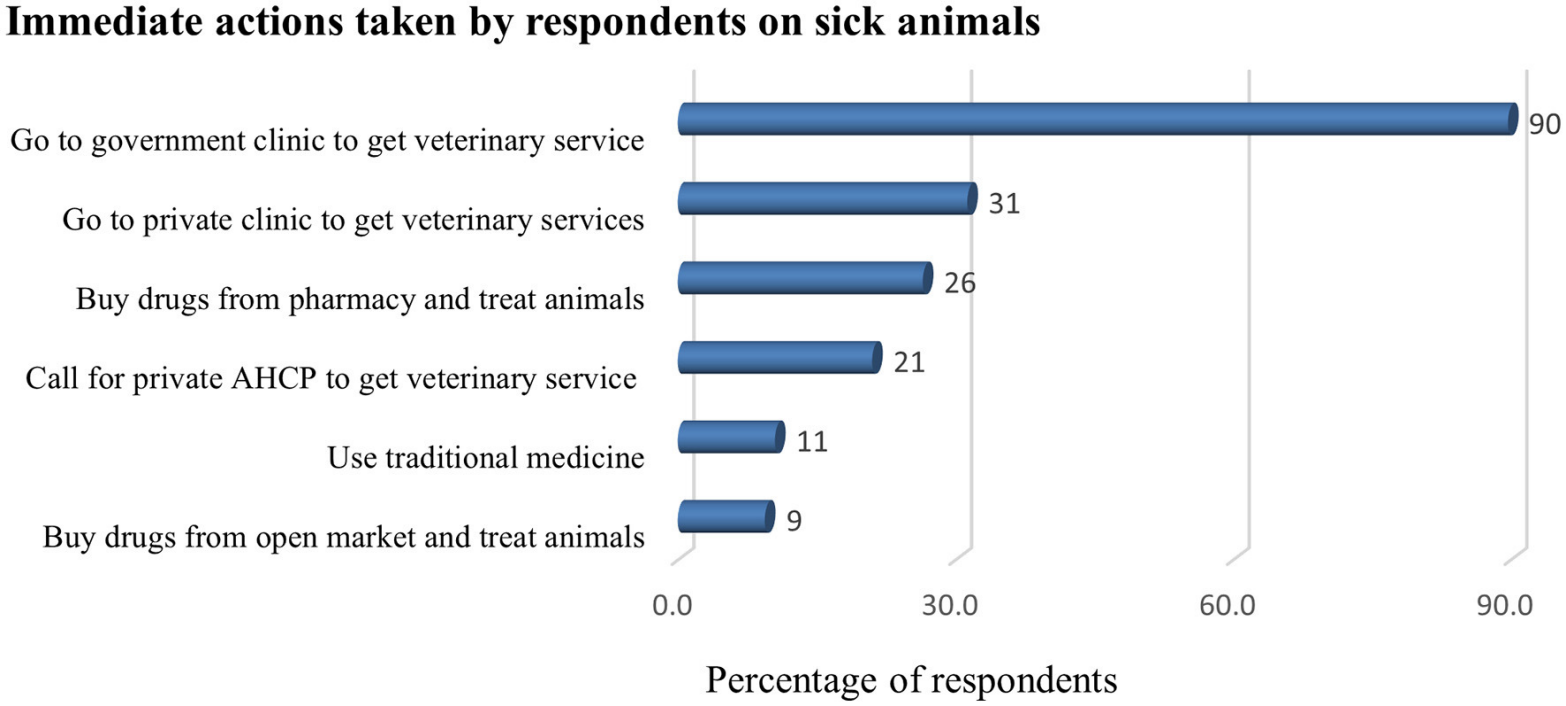
Actions taken by farmers (%) when their livestock get sick (*n* = 457). AHCP, animal healthcare provider.

The following drugs were used when treating sick animals: antimicrobials (28%), ethnoveterinary [traditional] medicines (i.e., application of veterinary folk knowledge, theory, and practice to treat ailments of livestock; 24%), and anthelmintics (9%). Antibiotics (21%) and trypanocides (11%) were the most used antimicrobials. Most drugs were self-prescribed and bought from a pharmacy (27%) or bought from a shop or open market (9%; Figure 3). Vaccination was reported by 13% of the farmers as a preventive measure and 56% practiced vaccination. Farmers vaccinated their animals against the following major livestock diseases: blackleg (53%), pasteurellosis (35%), LSD (25%), CBPP (20%), sheep pox (18%), FMD (22%), and anthrax (0.4%).

The results showed that 88% of livestock producers agreed with the statement that they need consultations or regular visits by animal healthcare professionals (AHCP) to their animals. Among respondents involved in this survey, only 9% have a regular AHCP and 6% have health/medicine use records at their farms. The non-availability of AHCP in the area (58%) followed by the high cost of therapy (25%) and a lack of need to have visits by AHCP (7%) were the main reasons why the farmers do not have regular supervision.

### 3.3. Knowledge, attitude, and behavior of farmers on AMU, antibiotic residues, and AMR in livestock production

#### 3.3.1. Knowledge

Despite farmers reporting a high level of familiarity with vaccines (92%), only 32% of them correctly explained the use of vaccines for disease prevention (Table 3). Moreover, there was a low level of understanding of antibiotics or antimicrobials (7.0%). Furthermore, 17% of the respondents were aware of the drug withdrawal period. Only 5% of respondents knew about drug residues, and only 3% gave a correct explanation of what drug residues mean (defined as the presence of veterinary pharmaceutical products such as antimicrobials and deworming products in milk, meat, or other animal products) and how it occurs. An assessment of livestock producers regarding the primary sources of information through which they had acquired awareness about drug residues for the first time showed that very low percentages were from veterinary clinicians who treated sick animals (2%), other veterinary professionals working in the clinic (1%) and mass media (0.2%). Finally, only a few livestock producers (9%) had heard of AMR or antibiotic resistance.

**Table 3:** Farmers' knowledge score about questions related to antibiotic use, resistance, and residues on 457 livestock farms in central and western Ethiopia, 2018.

**Question about knowledge (desirable answer)**		**Bako**	**Nekemte**	**Sebata**	**Overall correct answer**
		**(*N* = 185)**	**(*N* = 117)**	**(*N* = 155)**	
		***n*** **(%)**	***n*** **(%)**	***n*** **(%)**	***n*** **(%)**
K1. Do you know what vaccines are/do? (Correct^a^)		25 (14)	41 (35)	81 (52)	147 (32)
K2. Do you know what antibiotics or antimicrobials are/do? (Correct)		4 (2)	27 (23)	1 (1)	32 (7)
K3. Do you know what antibiotic residues are/do? (Correct)		0 (0)	13 (11)	0 (0)	13 (3)
K4. Do you know what drug withdrawal means? (Correct)		18 (10)	53 (45)	6 (4)	77 (17)
K5. Do you know what antibiotic resistance or AMR is/do? (Correct)		2 (1)	34 (29)	3 (2)	39 (9)
The overall level of knowledge score^b^	Good	0 (0)	6 (5)	1 (1)	7 (2)
	Moderate	0 (0)	22 (19)	0 (0)	22 (5)
	Poor	185 (100)	89 (76)	154 (99)	428 (94)
	Sufficient^*^	0 (0)	28 (24)	1(0.6)	29 (6)

^a^Correct means a desirable answer with a detail explanation to a given question. For instance, the answer for K1 “vaccines are used for disease prevention.”

^b^Score between 80 and 100% was categorized as good, 50 and 79% as moderate, and <50% as poor. The final knowledge score was dichotomized and those answers ≥50% correct (score of both good and moderate level of score) in a knowledge section of the questionnaire were considered to have sufficient knowledge while those answers <50% correct (score of poor) were considered to have insufficient (poor) knowledge, respectively.

^*^Both good and moderate levels of knowledge scores were considered as sufficient knowledge; %, percentage; n, frequency of desirable answer.

Overall, most participants (94%) had insufficient (poor) knowledge about AMU, drug residues, and AMR (Table 3). The knowledge score of farmers from the three sites showed statistically significant differences (*p* < 0.001), where all farmers from Bako (100%), Sebata (99%), and Nekemte (76%) had insufficient (poor) knowledge about AMU, residues, and AMR.

#### 3.3.2. Attitudes regarding AMU and AMR

The highest number of positive attitudes were about the statement that “good farm hygiene and proper feeding could be a solution to curb AMR” (94%) followed by “the relevance of getting consultation from veterinarians or AHCP regarding their animal health management and AMU” (88%). More than half of the respondents did not agree with developing new medicines/vaccines as a possible solution to curb AMR (62%), and only very few of them agreed with the statement “using the wrong antimicrobials cannot cure sick animals” (6%; Table 4). The overall level of farmers' attitude score toward contributing factors for AMR indicated that most of them (97%) had unfavorable attitudes (Table 4).

**Table 4:** Attitude score of participants toward contributing factors for antimicrobial resistance on 457 farms in central and western Ethiopia, 2018.

**Question about attitudes (desirable answer)**	**Bako**		**Nekemte**		**Sebata**		**Overall correct answer**	
	**(*****N*** = **185)**		**(*****N*** = **117)**		**(*****N*** = **155)**			
	***n***	**%**	***n***	**%**	***n***	**%**	***n***	**%**
A1. Using the wrong antimicrobials cannot cure sick animals (Yes/Agree)	3	2	25	21	1	1	29	6
A2. It is important to get a consultation from a veterinarian before giving antimicrobials to animals (Yes/Agree)	170	92	101	86	130	84	401	88
A3. AMR can cause antimicrobials not to work properly or unable to cure sick animals (Yes/Agree)	4	2	21	18	3	2	28	6
A4. Poor adherence to treatment can cause antimicrobials not to work properly or unable to cure sick animals (Yes/ Agree)	3	2	29	25	2	1	34	7
A5. Animal owner's self-prescription may cause antimicrobials not to work properly (Yes/Agree)	3	2	22	19	2	1	27	6
A6. Poor quality medicine can cause antimicrobials not to work properly (Yes/Agree)	3	2	18	15	3	2	24	5
A7. Poor farm hygiene and animal overcrowding can contribute to antimicrobials not working properly (Yes/Agree)	3	2	7	6	1	1	11	2
A8. Poor animal feeding practices can cause antimicrobials not to work properly or not cure sick animals (Yes/Agree, strongly agree)	3	2	8	7	1	1	12	3
A9. Proper disease diagnosis and treatment might be a solution to curb AMR (Yes/Agree)	1	1	36	31	143	92	180	39
A10. Adhering to recommended drug withdrawal period could be a solution to curb AMR (Yes/Agree)	1	1	13	11	129	83	143	31
A11. Strict adhering to a proper waste disposal system could be a solution to curb AMR (Yes/Agree)	0	0	12	10	130	84	142	31
A12. Good farm hygiene and proper feeding could be solutions to curb AMR (Yes/Agree)	168	91	117	100	144	93	429	94
A13. Developing new medicine/vaccine could be a solution to curb AMR (No /disagree)	185	100	85	73	12	8	282	62
**The overall level of attitude score** ^a^								
Good	0	0	0	0	1	1	1	1
Moderate	3	2	11	9	1	1	15	3
Poor (not favorable)	182	98	106	91	153	99	441	97
Favorable^*^	3	2	11	9	2	1	16	4

^a^Score between 80 and 100% was categorized as good, 50 and 79% as moderate, and <50% as poor. The final scale agreement of attitude scores was dichotomized and those answers ≥50% correct (score of both good and moderate level of attitude score) were considered to have favorable attitude while those answers <50% correct (score of poor) were considered to have not favorable (poor) attitude, respectively.

^*^Both good and moderate were considered as favorable attitudes; n, frequency of desirable answer; %, percentage of desirable answer.

The farmers' perception regarding what causes “antimicrobials not to work properly or unable to cure sick animals” was also assessed. The result indicated that very few farmers perceived that poor adherence to correct treatment (7%), wrong use of antimicrobials (6%), AMR (6%), owner self-prescription (6%), poor feeding practices (3%), and animal overcrowding and poor farm hygiene (2%) are possible causes.

**Figure 4:**
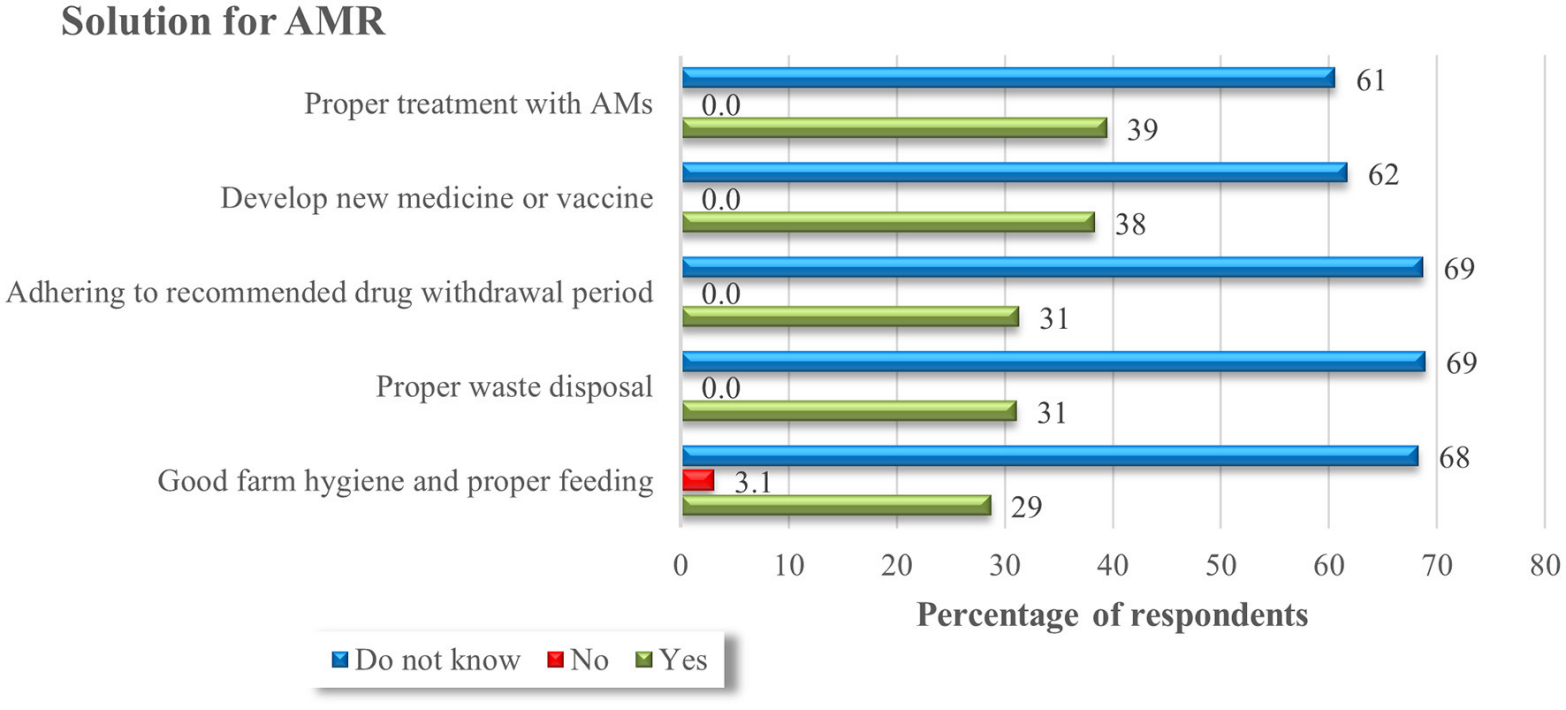
Farmer's perception of solution for AMR (*n* = 457).

Farmers' perception of the solution for AMR was also assessed. Figure 4 shows the possible solutions for AMR perceived by livestock producers in the study area.

#### 3.3.3. Behavior/practices regarding AMU

For a question to rank the top five antimicrobials used by farmers, “which drugs /antimicrobials/antibiotics are commonly used?” indicated that antimicrobials were the most used drugs (47%), followed by anthelmintics (43%) and acaricides (3%). Of antimicrobials, antibiotics were the most used veterinary drugs reported by 34% of livestock producers. Among antibiotics, oxytetracycline (28%), penicillin (28%), Pen-Strep [penicillin and streptomycin fixed combination] (18%), and sulfa drugs (0.06%) were reported as the five most used drugs in the study area.

Almost half of the livestock producers in the study area self-prescribed antimicrobials in the month before the interview (43%). They used these antimicrobials for cattle (41%), sheep (7%), goats (3%), and chicken (2%). The primary reason for AMU on their farms was “to treat sick animals or infection treatment” (21%), followed by “infection prevention or prophylaxis” (14%) and “both infection prevention and treatment” (9%) while more than half of the livestock producers do not use antimicrobials on their farms (57%). The current study also revealed that high use of antimicrobials was observed in ruminant-rearing farms (67%), followed by keeping ruminants together with chicken (44%) and chicken alone (33%) with statistically significant differences (*p* = 0.000). Species-based AMU analysis revealed that higher use was recorded in sheep rearing (62%, 37/60), followed by cattle (56%, 251/446), goats (46%, 21/46), and chicken (44%, 93/211) farms.

The assessment of the sources of antimicrobials by farmers showed that it was prescribed by AHCP (25%), self-selected (20%), and recommended by colleagues/neighbors (1%; Supplementary Table 1). Most livestock producers reported that they obtained antimicrobials from veterinary clinics (71%) and veterinary pharmacies or drug vendors (55%). However, about one-tenth of them also got drugs from the open market (9%; Supplementary Table 1).

In most cases, AHCPs (82%) and animal owners or farm supervisors (14%) were responsible for giving the drugs to the patient. Over the last 1-month period before the interview, more than half of the total farms used antimicrobials at least once (53%) or more than two times (18%). This study also noted that 24% of the total respondents self-prescribed antimicrobials for their neighbors (Supplementary Table 1).

The response to the question related to whether farmers practicing of stopping to give antimicrobials to their animals before the intended duration of therapy or not showed 35% of them stopped before the intended period. The primary reason was believing the animal was cured of the disease (25%), followed by reserving the drug for later use (7%), due to no observed clinical improvements (4%) and the inability of the patient to walk to distant veterinary clinics (2%). Farmers were also asked to answer their practices of administering a full dose of antimicrobials for the recommended period. Their response indicated that 31% did not give a full dose of drugs to the sick animals with the main reasons they believed that the treatment is sufficient (25%), the absence of sufficient money to buy the remaining drug or to pay for veterinary services (13%), advised by others (7), and the absence of AHCP at nearby (2%).

Overall, ~80% of the participants had good behavior about AMU and 20% had bad behavior (Table 5).

**Table 5:** Behavior score of livestock producers related to antimicrobial use on 457 farms in central and western Ethiopia, 2018.

**Question about behavior/practices (desirable answer)**	**Bako**		**Nekemte**		**Sebata**		**Overall good behavior**	
	**(*****N*** = **185)**		**(*****N*** = **117)**		**(*****N*** = **155)**			
	***n***	**%**	***n***	**%**	***n***	**%**	***n***	**%**
P1. Do you self-prescribe antimicrobials on your farms? (No)	150	81	13	11	96	62	259	57
P2. Do you self-prescribe antimicrobials on your farms to treat sick animals (curative use only)? (No)	150	81	25	21	147	95	322	71
P3. Do you self-prescribe antimicrobials on your farms to prevent animals from infection (prophylactic use only)? (No)	182	98	73	62	98	63	353	77
P4. Do you self-prescribe antimicrobials on your farms to treat and prevent animals from infection (both curative and prophylactic use)? (No)	182	98	85	73	151	97	418	92
P5. Do you give antimicrobials to all animals when one is sick (metaphylaxis)? (No)	182	98	73	62	99	64	354	78
P6. Do you self-prescribe antimicrobials to neighbors? (No)	167	90	47	40	135	87	349	76
P7. Do you give a full dose of Antimicrobials to animals as per recommendation? (Yes)	163	88	37	32	115	74	315	69
P8. Do you stop using full-dose antimicrobials before the intended duration of therapy? (No)	159	869	20	17	120	77	299	65
P9. Do you stop using full dose antimicrobials believing the animal is cured or because it has improved? (No)	170	92	34	29	138	89	342	75
P10. Do you stop using full-dose antimicrobials for later use? (No)	178	96	94	80	152	98	424	93
P11. Do you use expired medicines? (No)	174	94	36	31	154	99	364	80
P12. Do you share antimicrobials prescribed for one animal with another? (No)	173	94	4	3	131	84	308	67
P13. Do you buy antibiotics without prescription paper? (No)	169	91	62	53	132	85	363	79
P14. Do you buy medicines from an open market? (No)	178	96	84	72	153	99	415	91
P15. Do you use animal products [milk, eggs, and meat] obtained from animals under antimicrobials therapy? (No)	12	7	19	16	4	3	35	8
**The overall level of behavior score** ^a^								
Good	155	84	3	3	73	47	231	51
Moderate	26	14	29	25	81	52	136	30
Poor (bad)	4	2.	85	73	1	1	90	20
Desirable (good) behavior^*^	181	98	32	27	154	99	367	80

^a^Score between 80 and 100% was categorized as good, 50 and 79% as moderate, and <50% as poor. The final scale agreement of behavior scores was dichotomized and those answers ≥50% correct (both good and moderate level of behavior score) were considered to have desirable (good) behavior while those answers <50% correct (score of poor) were considered to have undesired (bad) behavior, respectively.

^*^Both good and moderate level of behavior scores were considered as desirable (good) behavior; N, number of respondents; n, frequency of desirable answer; %, percentage of desirable answer.

### 3.4. Association of demographic characteristics and livestock diseases with KAB scores

The results of the relationship between demographic characteristics with the participants' KAB scores about AMU, antibiotic residues, and AMR are presented in Supplementary Table 2. Univariate analysis on “KAB” indicated that 94, 97, and 20% of the respondents had insufficient knowledge, unfavorable attitudes, and bad behavior, respectively.

Table 6 shows significant results in three final models from a multivariable analysis on knowledge about AMU, residues and resistance, attitude toward contributing factors to AMR, as well as the behavior of farmers around contributing factors to AMR. Our multiple logistic regression analysis showed that education level, farming experience, and farm type were positively associated with increased levels of respondents' knowledge of AMU, residues, and AMR (Table 6). For instance, respondents with college/university and high school education were ~10.6 and 5.5 times more likely to demonstrate sufficient knowledge than those without formal education (*p* = 0.002, OR_adj_ = 10.57, CI = 2.36–47.2; *p* = 0.007, OR_adj_ = 5.48, CI = 1.57–19.07), respectively. The farming experience was also significantly correlated with the respondents' knowledge score, with farmers who had more than 15 years of experience found to have sufficient knowledge compared with those who had <5 years of farming experience (*p* = 0.027, OR_adj_ = 28.69, CI = 1.45–567.46). Knowledge also increased with rearing chicken (*p* = 0.040, OR_adj_ = 22.36, CI = 1.14–436.56) or keeping both ruminants and chicken (*p* = 0.004, OR_adj_ = 4.42, CI = 1.6–12.17), compared with keeping only ruminants. However, the family size was correlated with a decreased level of knowledge, with farmers who had a larger family size of more than three children being found to have less knowledge than those who had three or fewer children (*p* = 0.000, OR_adj_ = 0.13, CI = 0.05–0.38; Table 6).

**Table 6:** Multivariate logistic regression model of livestock producers' knowledge, attitude, and behavior.

**Final generalized linear models**	**Coefficient**	**OR_adjusted_**	**95% CI**	***p*-value**
**Model 1. Factors associated with sufficient (good) knowledge about AMU and AMR**				
**Education**				
No formal education	Ref			
Primary	0.61	1.85	0.58–5.90	0.297
High school	1.70	5.48	1.57–19.07	**0.007**
College or university	2.35	10.57	2.36–47.20	**0.002**
**Farming experience**				
< 5 years	Ref			
5–15 years	2.33	10.29	0.55–192.56	0.119
>15 years	3.35	28.69	1.45–567.46	**0.027**
**Family size (number of children)**				
0–3	Ref			
≥4	−1.96	0.13	0.05–0.38	0.000
**Farm type**				
Ruminants^*^	Ref			
Chicken	3.10	22.36	1.14–436.56	**0.040**
Ruminant^*^ and chicken	1.48	4.42	1.60–12.17	**0.004**
**Model 2. Factors associated with favorable attitudes about AMU and AMR**				
**Education**				
No formal education	Ref			
Primary	0.86	2.36	0.45–12.35	0.307
High school	1.48	4.39	0.71–26.96	0.109
College or University	3.32	27.78	5.23–147.44	**0.000**
**Model 3. Factors associated with good behavior of AMU**				
**Knowledge score**				
Poor knowledge	Ref			
Having good knowledge level	−1.59	0.20	0.08–0.49	**0.000**
**Attitude score**				
Undesirable attitudes	Ref			
Having positive (desirable) attitudes	−0.80	0.44	0.12–1.55	0.205
**Farming experience**				
< 5 years	Ref			
5–15 years	−0.64	0.52	0.11–2.47	0.415
>15 years	−1.34	0.26	0.05–1.16	0.078
**Number of animal species reared**				
One	Ref			
Two	−0.78	0.45	0.24–0.86	**0.015**
≥Three	−1.93	0.14	0.06–0.31	**0.000**
**Number of diseases encountered in a year**				
Three or less	Ref			
Four or more	−1.03	0.35	0.16–0.77	**0.010**

CI, confidence interval; OR_adjusted_, adjusted odd ratio.

Values in bold indicate a significant difference with the reference category (p < 0.05).

^*^Indicates raising either of cattle, sheep, or goats or their combinations.

The analysis results also showed that only the educational level was strongly associated with the attitudes toward contributing factors to AMR, with respondents who attended college or university education being ~27.8 times more likely to demonstrate favorable attitudes (*p* = 0.000, OR_adj_ = 27.78, CI: 5.23–147.77) than those without formal education (Table 6).

The results also showed that the knowledge score, the number of animal species reared, and the number of diseases encountered on the farm in a year were strong determinants of respondents' behavior (Table 6). Farmers with sufficient knowledge scores were found to have less desired behavior compared with those who had poor knowledge (*p* = 0.000, OR_adj_ = 0.44, 95, CI = 0.12–1.55). Bad farmers' behavior was also strongly associated with farmers who are keeping three or more animal species (*p* = 0.000, OR_adj_ = 0.14, CI = 0.06–0.31) compared with keeping only one species. Similarly, the incidence of three or more diseases at the farm level was correlated with the decreased desired behavior of farmers about AMU (*p* = 0.010, OR_adj_ = 0.35, CI = 0.16–0.77) than those encountered with three diseases or fewer (Table 6).

The correlation analysis revealed positive linear correlations between the knowledge and attitude scores of the respondents (*r* = 0.1946, *p* = 0.000) and a weak negative correlation between knowledge and behavior scores (*r* = −0.2548, *p* = 0.000), and attitude and behavior scores (*r* = −0.1152, *p* = 0.0137).

## 4. Discussion

Inappropriate use of antimicrobials, especially antibiotics shared between humans and animals, plays an important role in the emergence of AMR (14, 36, 37). Livestock diseases are a priority problem for livestock keepers throughout Ethiopia and other low-income countries with a substantial livestock population and diversified climatic conditions that favor the presence of pathogens. Antimicrobials are widely used to manage various diseases (38–40). The livestock keepers' access to, use of, and satisfaction with animal health services significantly vary across livestock production systems, geographic locations, socioeconomic strata, and service providers (41). Varied resistance levels of drug-resistant bacteria have also been reported from livestock, farm environments, and farm employees, posing serious public health threats in low-income countries, including Ethiopia (42, 43). Hence, monitoring AMU in livestock provides useful information for policy development to mitigate AMR risks (44, 45). This study assessed livestock disease management practices and KAB of livestock producers from central and western areas of Ethiopia regarding AMU, antibiotic residues, and AMR. The study assessed the major contributing factors to AMU behavior in the livestock sector in Ethiopia, an example of a low-income country with large livestock populations, which, in turn, could contribute to AMR.

### 4.1. Farmers' livestock disease management practices

The current study showed that livestock keepers are aware of common infectious diseases of livestock in their area. More than half of the farms had vaccinated their animals against most endemic infectious diseases (presented in Table 2). Diseases that do not allow for vaccination, such as mastitis, trypanosomiasis, and bovine TB, were also reported from the study farms. Trypanosomiasis was reported from Bako and Nekemte areas with statistically significant differences across the three study sites (*p* ≤ 0.001). This could be due to variation in vector (tsetse flies) distribution (46). This is also consistent with a previous study (47) that the disease distribution could be potentially affected by variations in the AEZ of the country.

The current study shows that antimicrobials were the most widely used drugs by farmers. Most farmers relied on the veterinary services delivered by the government and private clinics. Farmers in the study area mostly take sick animals to veterinary clinics or did consult animal health professionals for their drugs, while they also use alternative traditional medicines (Figure 3). Moreover, farmers used antimicrobials without a prescription to treat sick animals. This study agrees with similar studies conducted in Ethiopia (20, 21, 23). The habit of farmers getting professional consultancy for their drug use could contribute to improving their AMU behavior. However, their habit of buying veterinary drugs from the open market (Supplementary Table 1) and the involvement of community animal health workers, i.e., farmers selected from the community and who have received limited animal health training, in the current study could contribute to the extensive misuse of antimicrobials in the livestock sector.

The antibiotics (21%) of oxytetracycline, Pen-Strep and penicillin, and trypanocides (11%) were the most widely used antimicrobials by farmers to manage livestock diseases. Ethiopian farmers can get access to these antimicrobials without a prescription (21, 23). This finding is supported by other similar studies conducted in Ethiopia (20, 21, 23, 48). The primary reason for AMU by livestock producers on their farms in the present study was “to treat sick animals” (21%), followed by “prophylactic use” (14%). Higher than our finding, livestock farmers in Vietnam (69%) (19), Ethiopia (42%) (23), and other African countries (38%) (49) confirmed that they mainly use antimicrobials for treatment purposes.

### 4.2. The knowledge of AMU, antibiotic residues, and AMR

A better understanding and good attitudes of farmers about vaccines, antimicrobials, antibiotic residues or drug withdrawal, and AMR are expected to reduce the risks contributing to AMR. Vaccines are used prophylactically to decrease the occurrence of the number of infectious disease cases, and thus antibiotic use and the emergence and spread of AMR (50). In the current study, only one-third of the farmers correctly explained the use of vaccines for disease prevention (32%), indicating most farmers do not understand why and when they use vaccines. Similarly, livestock producers had the same level of low understanding of the term “antibiotics” and “antimicrobials,” which is consistent with our previous study (20) but not in line with studies conducted in other LMICs (19, 51, 52).

Most livestock producers (94%) were not knowledgeable about AMU, AMR, and antibiotic residues; however, most of them had sufficient livestock farming experience and awareness of livestock diseases. The findings of the poor understanding of farmers about AMU and AMR were consistent with previous studies reported by the study mentioned in reference (19) (93%) in Vietnam, (53) (90%) Turkey, and (20, 22) (80%) Ethiopia. However, the current finding about the poor knowledge level of farmers is higher than the findings reported by the study mentioned in references (23) (50%) and (21) (70%) of livestock farmers in Ethiopia, who had poor knowledge about AMR. This study also showed that most farmers were not aware of drug residues, and <1-4th (17%) of farmers had a good understanding of what drug withdrawal meant. The current result is in line with reports from Ethiopia (21) and other African countries (54, 55). The poor understanding of livestock owners about drug residues and withdrawal periods is due to insufficient advice that the farmers get from veterinary clinicians and drug dispensers, and the absence of regular information-sharing practices to farmers through the mass media or other available outlets.

The knowledge gaps in appropriate AMU in livestock farms have been extensively discussed (21, 23, 35, 51). The multivariate model analysis of the current study proves that the farmers' knowledge significantly varied by different demographic characteristics, such as respondents' level of education, farming experience, farm type, and family size. The poor knowledge scores of the respondents in our current study could be associated with the low educational level of farmers, as more than one-third of the respondents had no formal education (39%) or attended only a primary school (42%). This study result agrees with the previous study conducted in Ethiopia (20, 21, 23, 56) and Sudan (57). The results were also in line with studies elsewhere (51, 52) in which the higher educational level of farmers together with more than 15 years of farming experience significantly increased the knowledge score on AMR. This is due to long years of farming experience that could increase farmers' awareness of livestock disease management using drugs and its consequences. Our study also revealed that the farm type, specifically either rearing chicken or ruminants together with chicken, was significantly associated with a higher knowledge score about AMU, residues, and AMR. This could be due to farmers mostly engaging in chicken production, getting supplementary training on chicken disease management and biosecurity, as higher AMU practices were observed in ruminant-rearing farms. Furthermore, the univariate analysis of the current study indicated that the knowledge score of respondents vary significantly across the study areas (*p* ≤ 0.001), where respondents from Nekemte had relatively less poor knowledge score (76.1%) than Sebeta (99.4%) and Bako (100%). This could be associated with a higher level of education (university graduate), as a higher number of graduates were recorded from Nekemte (10%) or an increase in farmers' awareness due to the repetitive occurrence of endemic livestock diseases, such as trypanosomiasis at the study farms. This is consistent with the previous findings from elsewhere (19, 51).

### 4.3. The attitudes toward AMU, antibiotic residues, and AMR

Almost all (97%) of the livestock producers in the present study had an unfavorable attitude score toward contributing factors to AMR. This is in line with a recent study conducted in Ethiopia (85%) (22). The farmers' perception regarding what causes “antimicrobials not working properly or unable to cure sick animals” indicated that most of the respondents do not know the reason why antimicrobials fail to cure sick animals (83%), demonstrating the farmers have undesirable attitudes toward AMU. Furthermore, our current study findings indicated poor level of farmers' perception of the solution of AMR (Figure 4). However, most farmers have positive attitudes toward the statement about “good farm hygiene and proper feeding could be a solution to curb AMR” (94%), as they believe that proper feeding is the remedy to all problems including the prevention of infections. This wrong perception of farmers regarding risk factors for AMR could potentially facilitate the emergence and spread of AMR organisms in the livestock sector.

Farmers' access to sufficient advice from animal health experts about how to use veterinary drugs will minimize the misuse of antimicrobials on farms. In the current study, most respondents agreed on the importance of getting a consultation from AHCPs before using antimicrobials on animals (88%). Comparable to our findings, the study conducted on livestock producers in Vietnam (95%) (19), and other African countries (74%) (49) agreed with the significance of professional advice on prudent AMU. However, another study conducted in Ethiopia reported 39% of farmers agreed on the relevance of advice by AHCPs on AMU (22). Furthermore, our study indicated that farmers mostly use milk obtained from a cow under antimicrobial therapy either for home consumption or other purposes (Supplementary Table 1). This is a result of having prior information about the milk processing plant can impose a penalty on farmers who supply milk contaminated with antimicrobials, whereas the use of milk contaminated with antibiotics either for home consumption, selling to neighbors, or giving to calves is indicating a lack of farmers' understanding of the harmful effects of antibiotic residues on humans and calves. This could also be associated with the absence of compensation for the discarded milk by the milk processing plants. Our study finding supports the justification for the inappropriate use of antimicrobials in animal production, especially in resource-poor countries, would be the main driver of AMR in both humans and animals. Moreover, the study warrants the relevance of counseling by drug dispensers and clinicians to aware farmers of antibiotic residues and improves their knowledge about prudent AMU. Compensating the poor farmers for the discarded milk would also minimize the risk of AMR associated with the exposure of humans and animals to low concentration of antibiotics in the milk as residues.

The outcome of the multivariate model analysis of this study indicates that the farmers' attitude score was solely influenced by their level of education. The result is in line with (51, 52) where the higher educational level of farmers significantly increased the attitude score on AMR.

### 4.4. The behavior of livestock producers on AMU and AMR

The farmers' self-prescribing practices, while they lack detailed knowledge about the disease etiology and drug selection, could result in the administration of subtherapeutic doses or the use of unnecessary antimicrobials. In the present study, ~½ of the study farms (53%) use antimicrobials at least once per month, and farmers administered medicines to their animals by themselves (14%). The study also demonstrated that one-third of farmers share the antimicrobials with other animals and also reserve them for later use. In the worst scenario, a quarter of them keep expired medicines on their farms and use them when needed. Extensive misuse of antimicrobials could increase selection pressure for the emergence of AMR, as supported by previous studies (58–60), and could have negative health and economic impacts. Our study finding supports the justification indicated by studies, accessing antimicrobials without prescription, and fragmented governance of AMU in animal production are the main drivers of AMR (6). Furthermore, in the current study, farmers stopped giving antimicrobials to their animals before the intended duration of therapy for various reasons, as previously reported by the study mentioned in reference (21, 23), which supports our findings. Not administering the recommended dose for the intended duration could be the main driver of AMR in animal production, as indicated by a previous study (61).

Good or favorable AMU behavior in livestock production, i.e., proper use of antimicrobials by professionals for the management of livestock diseases, contributes to maximizing therapeutic efficacy and minimizing the selection of resistant micro-organisms. The overall AMU behavior score of livestock producers in this study was favorable (80%) while 20% of the respondents had a poor (bad) level of behavior score points. Farmers in the group “favorable” have some improper practices as well. Greater than these findings, other studies conducted in Ethiopia showed livestock farmers had improper AMU practices (79%) (22), (72%) (21), and 53% (23). The variations in the farmers' AMU practices could be associated with their better understanding of endemic livestock disease management, the availability of improved veterinary services or treatment options, or the methodological differences used to assess the farmers' behavior.

The multivariate model analysis of the current study showed that having good knowledge, keeping two or more animal species, and the occurrence of four and more livestock diseases on the farm in a year were strongly associated with the bad behavior of farmers about AMU (*p* < 0.05). The repetitive occurrence of livestock diseases on a farm would increase farmers' awareness about the disease and its management practices. A high disease incidence may also be used as an opportunity for farmers to observe and learn from AHCPs about how the case is handled: drug selection, duration of therapy, and route of drug administration to the patient. In this scenario, however, the rational use of drugs by the farmers could be affected by various factors, such as level of understanding, the appropriate use of drugs by the AHCP, drug availability, and the presence of cases having similar signs and symptoms. Previous studies on the KAB of farmers on AMU and AMR revealed that farmers' knowledge and attitudes are essential for effecting changes in behavior (22, 23). However, surprisingly, our investigation revealed a negative correlation between respondents that has sufficient knowledge and desired attitudes with poor (bad) AMU behaviors. This study revealed those farmers who have relatively a better understanding of antimicrobials with a high level of education had bad behavior about AMU in livestock disease management. This could be due to the fact that educated farmers and/or farmers with more years of farming experience may have a better understanding of livestock disease and practice self-administration of available antimicrobials to sick animals. This was often practiced by chicken producers, pastoralists, and farmers residing in tsetse belt areas of the country. In rural parts of Ethiopia with limited veterinary services, it is very common that most farmers (dominantly male) with the ability to read or understand English, or with some formal education, or a family head practice self-prescription of antimicrobials. Upon communication with the drug dispensers in the area, these farmers administer antimicrobials to sick animals or use antimicrobials for prophylaxis to prevent infections. This problem is further worsened by the weak veterinary drug regulatory system that legally enforces professionals not to sell antimicrobials without a prescription.

In general, our study indicates that the AMU behavior of the farmers in a resource-poor country with a large livestock population, such as Ethiopia, could be highly influenced by poor knowledge and undesired attitudes about AMU, antibiotic residues, and AMR. These findings support that the report of misuse or overuse of antimicrobials in animals (62) and lack of knowledge about prudent antibiotic use and AMR (63) are the most important factors for the development and spread of AMR. This suggests the need for improving the farmers' KAB regarding prudent AMU in livestock farming systems to minimize the risks of AMR.

#### 4.4.1. Study limitation

This study has some limitations. Primarily, a few questions were used in the final questionnaire tool in assessing the knowledge of the respondents because the farmer's response to most of the knowledge questions during the piloting phase was either “no” or “I do not know.” Second, the quality of the data generated from the interview might have been affected by recall bias. However, to minimize the bias about AMU behavior, it was assisted by using a demonstration box containing major veterinary drugs (injectable antibiotics, anthelmintics, trypanocides, and vitamins). Third, livestock diseases self-reported among the participants might have also affected the type of disease diagnosis as most disease names were described either by their local names or in their clinical presentations. Nevertheless, misdiagnosis is possible, given that it is only clinical. To minimize the bias, the interpretation of the clinical symptoms into the disease name was carried out by data enumerators from the veterinary profession selected and trained in the local area. Lastly, the geographical representation of the study areas, starting from the most central to the peripheral part to the western route, was selected with variations in veterinary services, AEZ, and disease distribution. The study included samples from the most central (Sebata), western (Nekemte), and in-between central and western parts of the country (Bako). The findings of the current study might not represent the whole country as the country has many more AEZ with more diversified disease distribution and accessibility to veterinary services.

## 5. Conclusion

Our current study findings contribute essential information for improving and implementing prudent AMU behavior in the livestock sector in resource-poor countries with large livestock populations. Farmers in the study area have poor knowledge and undesired attitudes toward AMU, antibiotic residues, and AMR. On the contrary, they have relatively good livestock disease management practices and AMU behavior. Their KAB toward AMU and AMR is linked to several factors, particularly level of education, farm type, farming experience, livestock disease incidence, and the number of animal species reared. Farmers with higher levels of education are linked to good knowledge and more favorable attitudes. Conversely, a higher level of education and high farming experience are highly linked to bad AMU behavior. Farmers included in this study are aware of livestock diseases and reported several varied reasons for AMU on their farms, mainly to treat infection and for prophylactic use. The findings of the current study provided baseline evidence about the KAB of livestock producers from a developing country with an increasing livestock population. This suggests the need for intervention measures to improve the farmers' KAB regarding proper animal disease management, prudent AMU, and restrictions on purchasing antimicrobials without prescription to curb AMR.

## Data availability statement

The original contributions presented in the study are included in the article/Supplementary material, further inquiries can be directed to the corresponding author.

## Ethics statement

The studies involving human participants were reviewed and approved by Institutional Board of Research Ethics Committee of the Addis Ababa University College of Veterinary Medicine and Agriculture (Ref. No: VM/ERC/01/06/10/2018). The patients/participants provided their written informed consent to participate in this study.

## Author contributions

TT and FR conceived the study and developed the methodologies and questionnaire. TT collected the field data and wrote the draft manuscript. TT, KA, and HH conducted the statistical analysis. KA, JS, and HH supervised the study and edited the manuscript. All authors listed have made a substantial, direct, and intellectual contribution to the work and approved it for publication.
