# Supplementary material for: Livestock producers' knowledge, attitude, and behavior (KAB) regarding antimicrobial use in Ethiopia
Source: Front Vet Sci. 2023 May 19;10:1167847. doi: 10.3389/fvets.2023.1167847 (PMC10235446; doi:10.3389/fvets.2023.1167847)
Supplement: Supplementary file 1 [file Data_Sheet_1.PDF]

Supplementary file 1: Questionnaire. Livestock disease management practices and knowledge, attitudes and behaviors of livestock producers regarding antimicrobial use, residues and resistance in Ethiopia

Code of respondent ________________________________
Farm name: ________________________________
Address (Tell): ________________________________
District /Town: ________________________________
Kebele/PA: ________________________________
Date: ________________________________Time: from________to_______

A. Sociodemographic characteristics of livestock owners/respondents

1. Which of the following best describes your gender?
   - Male
   - Female
   - Prefer not to answer
2. Which of the following best describes your age groups (in years)?
   - ≤ 30
   - 31-45
   - 46-60
   - ≥ 61
   - Prefer not to answer
3. Educational level:
   - Illiterate (No school /not able to read and write)
   - Primary level (grade 1-8)
   - High school (grade 9-12)
   - College Diploma
   - Degree (Professional)
4. Which of the following best describes your family size groups (number of children)?
   - 1= 0-3 child
   - 2= 4-6 child
   - 3= 7-10 child
   - 4= >10 child
   - 5= prefer not to answer
5. Were you raised on a livestock farm?
   - Yes
   - No
6. If yes to Q5, which of the following best describes your number of years in livestock farming?
   - <5 years
   - 5-15 years
   - >15 years
   - prefer not to answer
7. Which species, type and number of animals do you keep?

Species	Type	Number
○ Cattle	________________	_______________
○ Chicken	________________	_______________
○ Goat	________________	_______________
○ Camel	________________	_______________
○ Others (specify)	________________	_______________

B. Animal Health

1. What common livestock diseases do you know or are aware of them?

○ ________________________________<br>○ _________________________________<br>○ _________________________________<br>○ _________________________________<br>○ __________________________________<br>○ __________________________________	○ ________________________________<br>○ _________________________________<br>○ _________________________________<br>○ _________________________________<br>○ __________________________________<br>○ __________________________________

2. What are common livestock diseases on your farm and what type of preventive practices/medicines are used for each disease?

**Common livestock diseases/symptoms**		Often encountered on your farm?		What type of preventive practices/ medicines do you use?
		**Yes**	**No**	
○	Anthrax			
○	Blackleg			
○	Bovine TB			
○	Bloat			
○	Diarrhea or Dysentery			
○	Foot and mouth disease (FMD)			
○	Lumpy skin disease (LSD)			
○	Mastitis			
○	Pasteurellosis/Pneumonia			
○	Trypanosomosis			
○	CBPP			
○	Others (list down)			

3. On average, how often did you encounter illnesses in any of your dairy animals per month?
   - ○ Always
   - ○ Sometimes
   - ○ Rarely
   - ○ Never
4. What did you do when encountering such animals' illnesses? More than one response is possible.
   - ○ Call for private animal HCP/Vet
   - ○ Go to the government animal health clinic
   - ○ Go to a private vet clinic
   - ○ Go to a nearby veterinary pharmacy and buy medicines
   - ○ Go to any shop/open market and buy medicines
   - ○ Use traditional medicines in your area
   - ○ Slaughter the animal
   - ○ Other (specify) ________________________________

1. Do you have health/medicines use records of each animal? *(Behavior)*
   - ○ Yes
   - ○ No
2. Do you have a regular visiting animal healthcare provider?
   - ○ Yes
   - ○ No

If the answer is "No", why?

- Not needed
- The cost of treatment is expensive
- Not available in the area
- Other (specify)_______________

3. Do you know the use of vaccines for animals? *(Knowledge)*
    - Yes
    - No
4. If the answer is 'yes', what do vaccines do? *(mark as appropriate)*
    - Prevent animals from becoming sick ☐ Yes ☐ No
    - Cure sick animals ☐ Yes ☐ No
    - Both above ☐ Yes ☐ No
    - Others (specify)___________________________
5. Did you vaccinate your animals for the following diseases (see the list in the table below)?

Disease	Vaccinated (Yes/No)	When? (Season)	How many times per year?
○ Anthrax			
○ Blackleg			
○ Pasteurellosis			
○ Foot and mouth disease (FMD)			
○ Lumpy skin disease (LSD)			
○ Sheep /goat pox			
○ CBPP			
○ Others (list down)			

B. Antimicrobials/Antibiotics Use, Residues, and Resistance

1. Do you know what antimicrobials mean? (check by e.g. in the box of drugs) *(Knowledge)*
    - Yes (correct explanation- drugs that are used to treat or prevent infectious diseases caused by bacteria, fungi, protozoans, and virus; supported by e.g.)
    - No
2. Do you know what antibiotics mean? (check by e.g. in the box of drugs) *(Knowledge)*
    - Yes (correct explanation- drugs which are used to treat bacterial infections; supported by e.g.)
    - No
3. Have you ever self-prescribed antimicrobials for your animals in the last month? *(Behaviour)*
    - Yes
    - No
4. If yes to Q3, for which food animals most often do you use antimicrobials/antibiotics?
    - Cattle
    - Sheep
    - Goats
    - Chicken
    - Others (specify) _____________________
5. If yes to Q3, why do you use antimicrobials/antibiotics? Because I wanted to
    - Treat the sick animal
    - Prevent (prophylactic/metaphylaxis) from being sick
    - Both treatment and prophylactic use

- Others (specify) ___________

6. If yes to Q3, from where are the medicines used?
    - Prescribed by a healthcare provider
    - Self-selected
    - Recommended or given by neighbours
7. Who is administering the AMs? *(Behavior)*
    - Veterinarians (animal health professionals)
    - Animal owners (non-vet)
    - Farm supervisor (non-vet)
    - Other (specify)_________________
8. Were any of the animals had any adverse medicines events/reactions during administration of the medicines (such as hives, collapsing, abortion, decrease in milk production, fever, lethargy, respiratory distress, infertility, lumps or swelling in the injection area, medicines do not work as expected, etc)?
    - Yes
    - No
9. How frequently do you use antimicrobials/antibiotics over the one month? *(Behaviour)*
    - Once
    - 2-5 times
    - More than 5 times
10. Which drugs/antimicrobials/antibiotics are commonly used? (rank the top 5)
    - _____________________________(1st)
    - _____________________________(2nd)
    - _____________________________(3rd)
    - _____________________________(4th)
    - _____________________________(5th)
11. What are the sources of antimicrobials you used? (check all that apply). *(Behaviour)*
    - Open market/ Any shop
    - Human pharmacies
    - Veterinary pharmacies/drug shops
    - Veterinary clinics
    - Traditional practitioners
    - Community animal health worker
    - Others specify_____________________
12. Who advised you or provided information to administer antimicrobials/antibiotics? (check all that apply).
    - Veterinarian or animal health worker
    - Veterinary Pharmacy
    - Human pharmacy professional in Pharmacy
    - Pharmaceutical or feed distributor
    - Traditional medicine practitioner
    - Other farmers
    - Own experience
13. Have you ever self-prescribed antimicrobials for your neighbours' sick animals? *(Behavior)*
    - Yes
    - No
14. Do you administer the full dose and course of the antimicrobials as recommended? *(Behavior)*
    - Yes
    - No
15. If No to Q14, why not? *Multiple responses possible*

- Have no sufficient money ☐ Yes ☐ No
- Believed that it is sufficient ☐ Yes ☐ No
- Advised by others ☐ Yes ☐ No
- Others (specify) _______________

16. Do you ever stop giving antimicrobials before you were supposed to? (*Behaviour*)
    - Yes
    - No
17. If Yes to Q16, why did you stop giving antimicrobials? (*Behaviour*)
    - Because the antimicrobial does not work
    - Believed that the animal has improved or cured
    - To save for later use
    - Others (specify)_____________________________
18. Do you share the antimicrobials of one animal with another animal? (*Behaviour*)
    - Yes
    - No
19. Do you reserve antimicrobials for later use other than the current use? (*Behaviour*)
    - Yes
    - No
20. If the antimicrobials in your hands or those for some reason bought are expired, what do you do with them? (*Behaviour*)
    - I will use them when needed
    - I will not use them
    - Throw away
    - Return to where you bought
    - Other, specify
21. Do you think it is important to get a consultation with a veterinarian before giving antibiotics to the animals? *(Attitude)*
    - Yes
    - No
22. Have you heard /know about antibiotic/drug residues? (*Knowledge*)
    - Yes
    - No
        a. If Yes to Q 22, would you explain it? ____
           Correct explanation ☐ Yes ☐ No
        b. If yes to Q 22, how does it occur? ____
           Correct answer ☐ Yes ☐ No
        c. If yes to Q 22, where do you first learn about it? ____
23. Are you aware of the term "drug withdrawal period"? (*Knowledge*)
    - Yes
    - No
24. Did the animal healthcare provider (he/she) tell you the withdrawal period of the drug used and not to use the dairy products until the end of the withdrawal period? *(Behavior)*
    - Yes
    - No
25. What do you do with the milk obtained from the cow under antibiotic/antimicrobial treatment? *(Behavior)*
    - Discarding
    - Giving to calves
    - Selling to the neighbour/contractors
    - Use for home consumption
    - Sending to the milk processing plants in a separate container

- Other, specify____

26. Would it matter to you if you eat meat or other animal products such as eggs and milk while the animal is taking medicines (that contain antibiotics)? *(Attitude)*
    - Yes
    - No
    - I don't know
27. Were milk samples tested for antibiotic residues from:
    - Fresh cows? ☐ Yes ☐ No
    - Individual cows recently treated with antibiotics? ☐ Yes ☐ No
    - Bulk tank—before processor pickup? ☐ Yes ☐ No
    - Other? (Specify: _______________________)
28. Have you heard /know about antimicrobial resistance (*failed to cure diseases*)? *(Knowledge)*
    - Yes
    - No
29. Have you heard /know about antibiotic resistance (*failed to cure diseases*)? *(Knowledge)*
    - Yes
    - No
      a. If Yes to Q29, would you explain it? ________________________________
         Correct explanation ☐ Yes ☐ No
30. What causes antimicrobials/antibiotics not to work (*not cure sick animals*)? *(Attitude)*
    - Antimicrobial resistance ☐ Yes ☐ No
    - Poor adherence to treatment ☐ Yes ☐ No
    - Use of poor-quality medicines ☐ Yes ☐ No
    - Using the wrong antimicrobials ☐ Yes ☐ No
    - Overcrowding and poor hygiene and sanitation of animals ☐ Yes ☐ No
    - Poor feeding practices ☐ Yes ☐ No
    - Owner's self-prescription of antimicrobials for their animals ☐ Yes ☐ No
    - Others (specify)________________
31. What do you think about the solutions for AMR (antimicrobials not working to treat infectious animal diseases)? *(Attitude)*
    - Hygiene, proper feeding of animals ☐ Yes ☐ No
    - Proper diagnosis and treatment with AMs ☐ Yes ☐ No
    - Use of quality or legal sources AMs ☐ Yes ☐ No
    - Proper waste disposal ☐ Yes ☐ No
    - Adhering to the drug withholding time ☐ Yes ☐ No
    - Develop new medicines/vaccines ☐ Yes ☐ No
    - Do not know ☐ Yes ☐ No
    - Others (specify) ____________________
32. Is the information about_____________available to you?
    a. Good dairy/poultry farm practice ☐ Yes ☐ No
    b. Prudent antimicrobial use ☐ Yes ☐ No
    c. Antimicrobial/antibiotic resistance ☐ Yes ☐ No
33. Do you have any additional comments about antimicrobials use, resistance and containment not covered above?____
