# Supplementary material for: Livestock producers' knowledge, attitude, and behavior (KAB) regarding antimicrobial use in Ethiopia
Source: Front Vet Sci. 2023 May 19;10:1167847. doi: 10.3389/fvets.2023.1167847 (PMC10235446; doi:10.3389/fvets.2023.1167847)
Supplement: Supplementary file 2 [file Table_1.DOCX]

**Supplementary Table 1**. Practices of livestock producers related to AMU on 457 livestock farms located in central and western Ethiopia

| Practice | % (n) of respondents (yes) |
| --- | --- |
| *How farmers obtain veterinary AMs* | |
| Prescription by AHCP | 25 (116) |
| Self-select | 20 (93) |
| Recommendation by colleague/ neighbor | 1 (5) |
| *Source of antimicrobials* | |
| Veterinary clinic | 71 (324) |
| Veterinary pharmacy | 55 (251) |
| Human pharmacy | 8 (35) |
| Open market | 9 (42) |
| Traditional healers | 5 (23) |
| Community animal health workers | 3 (14) |
| *Who gives drugs to the patient?* | |
| AHCP/Veterinarian | 82 (374) |
| Animal owner/farm supervisor | 14 (66) |
| Veterinarians and animal owner | 3 (15) |
| Traditional healer | 0.4 (2) |
| *Frequency of AMs use at farm level* | |
| Once over one month | 53 (244) |
| 2-5 times over one month | 18 (82) |
| >5 times over one month | 1 (5) |
| Once in 2-6 months | 2 (8) |
| Only when animals get sick | 13 (61) |
| Not used at all | 12 (54) |
| Do not remember | 1 (3) |
| *Self-prescribed AMs to neighbors?* | 24 (108) |
| *Who informed/ advised you about how to use AMs?* | |
| Veterinarian/AHCP | 75 (344) |
| Professional in the pharmacy | 35 (158) |
| Own experience | 5 (24) |
| Colleague /another farmer | 2 (9) |
| *Do you reserve AMs for later use?* | 34 (156) |
| *What do you do on expired drugs /AMs?* | |
| Do not use it | 45 (204) |
| Dispose of it | 42 (193) |
| Use it when needed | 20 (92) |
| Return it to where I bought it | 2 (9) |
| Do not check it | 0.2 (1) |
| *Actions to be taken on milk obtained from a cow under AMs therapy* | |
| Giving to calves | 74 (338) |
| Use for home consumption | 25 (114) |
| Discard | 12 (54) |
| Send to a processing plant or sell to neighbors | 2 (10) |
| Do not know | 0.2 (1) |

*Key: AMs, antimicrobials; AHCP, animal healthcare provider; n, frequency of desirable answer; %, percentage of desirable answer.*

**Supplementary Table 2.** Univariable analysis of the association of demographic characteristics and livestock diseases on KAB of respondents

| Variable | Category | N | Sufficient knowledge about AMU, residue, and resistance | | | Favorable attitude towards contributing factors for AMR | | | Desired behavior on AMU | | |
| --- | --- | --- | --- | --- | --- | --- | --- | --- | --- | --- | --- |
|  |  |  | **n (%)** | **OR [95% CI]** | **P-value** | **n (%)** | **OR (95% CI)** | **P-value** | **n (%)** | **OR (95% CI)** | **P-value** |
| District | Bako | 185 | 0 (100) | Ref |  | 3 (2) | Ref |  | 181 (98) | Ref | 0.000 |
|  | Nekemte | 117 | 28 (24) | 48.45[6.48-362.19] | **0.000** | 11 (9) | 6.29[1.71-23.07] | **0.005** | 32 (27) | 0.008[.002-0.02] | **0.000** |
|  | Sebata | 155 | 1 (0.7) | 1 | - | 2 (1) | 0.79[.13-4.80] | 0.801 | 154 (99) | 3.40[.37-30.77] | 0.276 |
| Age (years) | <30 | 82 | 4 (5) | Ref |  | 2 (2) | Ref |  | 75 (92) | Ref |  |
|  | 30-45 | 275 | 23 (8) | 1.77[.597-5.302] | 0.301 | 13 (5) | 1.98[.438-8.98] | 0.373 | 205 (75) | 0.27[.12-0.62] | **0.002** |
|  | >45 | 100 | 2 (2) | 0.39[.071-2.22] | 0.295 | 1 (1) | 0.40[.035-4.53] | 0.463 | 87 (87) | 0.49[.23-1.64] | 0.341 |
| Education | Illiterate | 178 | 5 (3) | Ref |  | 2 (1) | Ref |  | 148 (83) | Ref |  |
|  | Primary | 191 | 10 (5) | 1.91[.640-5.705] | 0.246 | 5 (3) | 2.36[.45-12.35] | 0.307 | 146 (76) | 0.65[.39-1.10] | 0.111 |
|  | Highschool | 63 | 8 (13) | 5.03[1.58-16.01] | **0.006** | 3 (5) | 4.4[.71-26.96] | 0.109 | 55 (87) | 1.39[.60-3.22] | 0.438 |
|  | College /University | 25 | 6 (24) | 10.92[3.04-39.21] | **0.000** | 6 (24) | 27.78[5.23-147.44] | **0.000** | 18 (72) | 0.52[.20-1.35] | 0.182 |
| Family size (number of children) | ≤ 3 | 73 | 11 (15) | Ref |  | 2 (3) | Ref |  | 57 (78) | Ref |  |
|  | ≥ 4 | 371 | 18 (5) | 0.28[.12-0.63] | **0.002** | 13 (4) | 1.28[.28-5.83] | 0.742 | 303 (82) | 1.25[.67-2.31] | 0.475 |
| Farm experience (years) | <5 | 47 | 1 (2) | Ref |  | 1 (2) | Ref |  | 45 (96) | Ref |  |
|  | 5-15 | 147 | 8 (5) | 2.65[.322-21.737] | 0.365 | 6 (4) | 1.95[.23-16.687] | 0.539 | 129 (88) | 0.32[.07-1.42] | 0.135 |
|  | >15 | 244 | 18 (7) | 3.66[.477-28.133] | 0.212 | 8 (3) | 1.55[.19-12.76] | 0.679 | 185 (76) | 0.14[.03-0.59] | **0.008** |
| *(Continued)* | | | | | | | | | | | |

| *Supplementary Table 2. (Continued)* | | | | | | | | | | | | |
| --- | --- | --- | --- | --- | --- | --- | --- | --- | --- | --- | --- | --- |
| Number of animal species reared | One | 209 | 6 (3) | Ref |  | 6 (3) | Ref |  | 186 (89) | Ref | |  |
|  | Two | 192 | 15 (8) | 2.87[1.089-7.55] | **0.033** | 8 (4) | 1.47[.50-4.32] | 0.483 | 151 (79) | 0.45[.26-0.79] | | **0.005** |
|  | Three or more | 56 | 8 (14) | 5.64[1.87-17.01] | **0.002** | 2 (4) | 1.25[.24-6.38] | 0.786 | 30 (54) | 0.14[.07-0.28] | | **0.000** |
| Farm type | Ruminant* | 246 | 6 (2) | Ref |  | 5 (2) | Ref |  | 217 (88) | Ref |  | |
|  | Chicken | 4 | 1 (25) | 13.33(1.20-147.53) | **0.035** | 1 (25) | 16.06[1.41-182.54] | **0.025** | 4 (100) |  |  | |
|  | Ruminant* and chicken | 207 | 22 (11) | 4.75(1.890-11.969) | **0.001** | 10 (5) | 2.44[.82-7.27] | 0.108 | 207 (100) | 0.32[.19-0.52] | **0.000** | |
| Number of diseases encountered in a year | ≤ Three | 143 | 3 (2) | Ref |  | 3 (2) | Ref |  | 134 (94) |  |  | |
|  | ≥ Four | 314 | 26 (8) | 4.21[1.25-14.15] | **0.020** | 13 (9) | 2.01[0.56-7.18] | 0.280 | 233 (74) | 0.19[0.09-0.39] | **0.000** | |
| Major livestock disease | Blackleg | 290 | 28 (10) | 17.74[2.39-131.62] | **0.005** | 14 (5) | 4.18[.94-18.64] | 0.060 | 203 (70) | 0.04[.013-0.137] | **0.000** | |
|  | Bovine-TB | 168 | 12 (7) | 1.62[0.70-3.78] | 0.256 | 9 (5) | 4.54[1.21-17.05] | **0.025** | 142 (85) | 0.62[.34-1.12] | 0.115 | |
|  | Bloat | 306 | 24 (8) | 2.87[.97-8.44] | 0.055 | 13 (4) | 2.01[0.28-7.17] | 0.281 | 234 (77) | 0.25[.12-0.50] | **0.000** | |
|  | LSD | 197 | 14 (7) | 2.06[.84-5.03] | 0.110 | 11 (6) | 3.25[1.01-10.38] | **0.046** | 161 (82) | 0.56[.32-0.97] | **0.040** | |
|  | Mastitis | 261 | 26 (10) | 20.68[2.78-153.8] | **0.003** | 14 (5) | 5.27[1.18-23.47] | **0.029** | 178 (69) | 0.023[.005-0.09] | **0.000** | |
|  | Pasteurellosis | 262 | 21 (8) | 7.10[1.64-30.70] | **0.009** | 11 (4) | 2.36[.65-8.61] | 0.191 | 201 (77) | 0.10[.04-0.26] | **0.000** | |
|  | Trypanosomiasis | 226 | 24 (11) | 5.37[2.01-14.33] | **0.001** | 13 (6) | 4.63[1.30-16.50] | **0.018** | 149 (66) | 0.11[.06-0.21] | **0.000** | |

*CI, Confidence interval; OR, Odds ratio; n, number; AMU, antimicrobial use; AMR, antimicrobial resistance; *Ruminant means cattle, sheep, and/ or goats; values in bold indicated a significant difference with the reference category (p < 0.05)*
